# Vaccination Adherence and Barriers Among Non-healthcare Workers and Volunteers During the Hajj

**DOI:** 10.7759/cureus.108694

**Published:** 2026-05-12

**Authors:** Mohammed E Shaybah, Osama Alsehli, Ali Al Harthi, Rami M Babukur, Baher Alwaraky, Mohammed Bafaqih, Muhab Masoud, Mohammed Alzhrani, Abdulghafur Kashgari, Abdualrahman Al-Asmari, Mokhtar M Shatla, Majed Obaid

**Affiliations:** 1 College of Medicine, Umm Al-Qura University, Makkah, SAU

**Keywords:** mass gathering, saudi arabia, the hajj, vaccination, volunteers

## Abstract

Background: Hajj is one of the world’s largest annual mass gatherings and is associated with increased risks of communicable diseases. While healthcare workers are subject to mandatory vaccination policies, non-healthcare workers and volunteers lack clear immunization requirements. This cross-sectional online survey assessed vaccination adherence, identified barriers, and generated evidence to inform the development of inclusive vaccination policies for non-healthcare workers and volunteers.

Materials and methods: A cross-sectional survey was conducted among 68 non-healthcare workers and volunteers during the 2024 Hajj season. A structured self-administered online questionnaire was used to collect data on sociodemographic characteristics, vaccination status, and perceived barriers. Given the small sample size (n=68), findings should be interpreted as exploratory.

Results: Survey results revealed low uptake of meningococcal (n = 11; 16.2%) and pneumococcal (n = 10; 14.7%) vaccines, moderate adherence to influenza vaccination (n = 34; 50.0%), and higher coverage for COVID-19 vaccination (n = 48; 70.6%). Knowledge level was a significant predictor of vaccination uptake (p < 0.05). Reported barriers included a lack of awareness of vaccination requirements, misconceptions about side effects, and limited access to vaccination services. Despite high recognition of infection risks, adherence remained suboptimal.

Conclusion: Vaccination adherence among non-healthcare Hajj workers and volunteers appeared insufficient in this exploratory study, particularly for meningococcal and pneumococcal vaccines. The observed patterns suggest that educational gaps, unclear policy guidance, and limited vaccine accessibility may contribute to suboptimal adherence in this population; however, these observations should be interpreted cautiously, given the small sample size and exploratory design. Strategies such as improving health communication, facilitating vaccine access, and clarifying official vaccination requirements may be worth exploring in future interventions, though firm policy recommendations cannot be drawn from the current data alone. Future research with larger and more representative samples is needed to substantiate these findings and provide a more comprehensive assessment of adherence patterns and barriers among this understudied group.

## Introduction

Each year, millions of pilgrims from across the globe travel to Mecca, Saudi Arabia, to perform the Hajj, one of the five pillars of Islam and the largest annual mass gathering. The Ministry of Hajj and Umrah has issued multiple safety and health legislations to ensure the safety and health of pilgrims. One of those legislations requires pilgrims, staff, and volunteers in healthcare to receive vaccinations for communicable diseases [1].

To mitigate these health risks, the Saudi Ministry of Health has established policies that include both mandatory and recommended immunizations for all pilgrims and healthcare workers and require COVID-19 vaccination to minimize infection and restore public health for all citizens and residents of Saudi Arabia [2]. While these policies aim to protect both pilgrims and the broader community, unfortunately, the policies are not clear regarding non-healthcare workers and volunteers. There is no clear legislation for them regarding training and vaccination before the Hajj season. With no policy issued from a higher legislative power upon non-healthcare workers/volunteers, adherence to vaccinations varies [3].

Studies have revealed that adherence to vaccination among healthcare workers is higher [4]. However, there is limited published evidence regarding vaccination adherence among non-healthcare workers and volunteers. Moreover, that is one of the barriers to achieving immunity. A significant amount of literature identifies multiple barriers that minimize vaccination adherence. Several barriers further compound this problem in the current information environment, and non-healthcare workers are increasingly susceptible to vaccine misinformation and misconceptions, disseminated via social media and online platforms [5]. Logistical challenges, including inadequate vaccine availability, prolonged waiting times, and insufficient local health infrastructure, restrict access for many workers [6]. Additionally, cultural attitudes, including scepticism toward vaccine safety and effectiveness, shape individual vaccination decisions, making targeted and culturally sensitive educational interventions essential [6].

Understanding these barriers provides insights into how public health policies can be adapted to better meet the needs of this population and improve vaccination rates. Previous research suggests that tailored educational initiatives can enhance awareness and positively influence attitudes toward vaccination [7]. Therefore, this study aimed to (1) estimate vaccination adherence levels among non-healthcare workers and volunteers during the 2024 Hajj season; (2) identify sociodemographic and knowledge-related determinants of vaccine uptake; and (3) characterize the barriers perceived by this population as obstacles to receiving recommended vaccines.

## Materials and methods

Research description

This study employs a cross-sectional survey design to assess vaccination adherence and identify barriers preventing non-healthcare workers and volunteers from receiving vaccinations during the Hajj. This approach was selected to collect quantitative data during the Hajj season, thereby providing a clear understanding of the current vaccination status and challenges.

Population and sampling

The targeted population consists of non-healthcare volunteers/workers who participated in the 2024 Hajj season. These individuals include both local and international security personnel, coordinators, drivers, and logistics staff who assist in various operational roles but are not involved in healthcare services. The study targeted a population for which limited baseline data are available. As such, a formal a priori sample size calculation was not performed. The study was not powered to detect small effect sizes or to support robust inferential subgroup analyses. Any subgroup comparisons presented are therefore exploratory in nature and should be interpreted with caution.

A structured, self-administered online questionnaire was distributed to the public to minimize bias in selecting participants. The questionnaire was active (January 2025 to July 2025). Participants who didn't fit the inclusion criteria were excluded from the sample. This method allowed for efficient data collection from many participants and was chosen due to the widespread use of email and social media platforms such as WhatsApp Messenger and X (formerly Twitter). Due to the operational restrictions associated with the Hajj season, on-site or paper-based data collection was not feasible, which limited the achievable sample size.

Inclusion Criteria of Participants

Aged 18 years or older; participation in the 2024 Hajj season in a non-healthcare volunteer or worker capacity; and provision of electronic informed consent prior to questionnaire completion.

The data were collected through a structured, self-administered online questionnaire adapted from instruments used by Goni et al. [8] and Bulkhi et al. [6], with modifications made to reflect the specific context of non-healthcare workers. No formal psychometric validation was performed for this adapted version; however, face validity was reviewed by two members of the research team prior to distribution. The questionnaire included four main sections: (1) demographics, (2) vaccination status, (3) motivations for vaccination, and (4) barriers to vaccination.

Data analysis

All statistical analyses for this study were conducted using SPSS version 28.0 (IBM Corp., Armonk, NY, USA). Descriptive statistics were used to summarize the participants' sociodemographic characteristics, vaccination status, knowledge, attitudes, and practices. Categorical variables were presented as frequencies and percentages. To explore associations between participants' characteristics and vaccine uptake (meningococcal, influenza, and pneumococcal), Fisher's exact probability test was used due to the small sample size and categorical nature of the variables. Questionnaires with incomplete responses were excluded from the analysis. No imputation was performed for missing data, and only complete responses were included in the final dataset. Participants' overall knowledge regarding vaccines and infectious disease risks was assessed using a structured set of items, with each correct answer awarded one point. The total knowledge score was calculated by awarding one point per correct answer and subsequently categorized into two levels -- poor and good knowledge -- based on a 60% cutoff point. In this study, participants answered a structured set of knowledge items covering vaccine requirements, infectious disease risks, and general awareness of vaccination during the Hajj. A score of 60% or above was considered indicative of adequate knowledge, as it reflects a participant's ability to correctly answer the majority of items and demonstrates a functional understanding of the vaccination context relevant to their role as non-healthcare Hajj workers or volunteers. Participants falling below this threshold were classified as having poor knowledge, suggesting insufficient awareness that may contribute to low vaccine uptake. This cutoff is consistent with its application in comparable KAP studies conducted in similar populations, including Mallhi et al. [9], who used the same threshold to classify influenza vaccine knowledge among university students in Saudi Arabia, supporting its appropriateness for this study's context. A p-value of less than 0.05 was considered statistically significant. Given the small sample size, all significant associations should be interpreted as exploratory. Multivariate analysis was not performed due to the limited sample size and the exploratory nature of the study.

Ethical considerations

Ethical approval was obtained from the Institutional Review Board (IRB) of Umm Al-Qura University (Approval Number: HAPO-02-K-012-2025-03-2590). Participants were informed of their rights, including anonymity, confidentiality, and the voluntary nature of participation. Informed consent was obtained electronically before participants completed the survey.

## Results

Results of participant information

The demographic characteristics of the 68 non-healthcare workers and volunteers participating in the Hajj showed that the majority were aged between 18 and 39 years (n = 51; 75.0%), followed by those aged 40 to 60 years (n = 14; 20.6%), and only a small proportion were over 60 years old (n = 3; 4.4%). Most participants were male (n = 58; 85.3%), while female participants represented a smaller group (n = 10; 14.7%). Regarding nationality, the majority were Saudi nationals (n = 53; 77.9%), and the rest were non-Saudi (n = 15; 22.1%). As for duration of work, half of the participants had worked for five to 10 years (n = 34; 50.0%), followed by those with more than 10 years of experience (n = 26; 38.2%), while a minority had less than five years of experience (n = 8; 11.8%). In terms of chronic diseases, most respondents reported not having any chronic conditions (n = 55; 80.9%), while 13 participants (19.1%) indicated that they do have chronic illnesses (Table 1).

**Table 1:** Demographic Characteristics of Non-healthcare Workers and Volunteers Participating in the Hajj (N = 68)

Variable	n	%
Age (years)		
18-39	51	75.0
40-60	14	20.6
>60	3	4.4
Gender		
Male	58	85.3
Female	10	14.7
Nationality		
Saudi	53	77.9
Non-Saudi	15	22.1
Hajj work experience		
<5 years	8	11.8
5-10 years	34	50.0
>10 years	26	38.2
Chronic medical condition		
Yes	13	19.1
No	55	80.9

Vaccine status

The vaccination status of the 68 non-healthcare workers and volunteers participating in the Hajj reveals variable levels of vaccine uptake. The highest coverage was observed for the COVID-19 vaccine, with 48 participants (70.6%) reporting having received it, while 20 (29.4%) had not. Influenza vaccination was evenly distributed, with 34 participants (50.0%) reporting receipt and 34 (50.0%) not vaccinated. In contrast, only 11 participants (16.2%) had received the meningococcal vaccine, and 10 (14.7%) had received the pneumococcal vaccine, leaving a large majority unvaccinated for these two infections (83.8% and 85.3%, respectively).

Vaccine knowledge and perception

The majority of participants demonstrated strong knowledge and awareness of vaccination and infectious disease risks during the Hajj. Most agreed that vaccines help prevent the spread of infectious diseases, and 64 (94.1%) supported the idea that vaccination should be a requirement for working during the Hajj. Additionally, 61 (89.7%) recognized the risk of infection with respiratory pathogens such as meningococcal, pneumococcal, COVID-19, and influenza during the Hajj season. There was also high awareness about the general health risks, as 62 participants (91.2%) agreed that respiratory microbes can cause serious complications, and 66 (97.1%) recognized that crowded places increase the risk of spreading such infections. However, knowledge about the mandatory nature of specific vaccines was considerably lower. Only 25 (36.8%) believed the meningococcal vaccine is mandatory for the Hajj workers, and even fewer acknowledged the requirement for pneumococcal (n = 22; 32.4%), COVID-19 (n = 33; 48.5%), and influenza vaccines (n = 33; 48.5%) (Table 2).

**Table 2:** Knowledge and Perceptions of Non-healthcare Workers and Volunteers Regarding Vaccination and Infectious Disease Risks During Hajj (N = 68)

Item	Yes (n)	Yes (%)	No (n)	No (%)
Vaccines help prevent the spread of infectious diseases among Hajj workers and pilgrims	63	92.6	5	7.4
Vaccination should be a requirement for work during the Hajj season	64	94.1	4	5.9
Hajj workers are at risk of infection with meningococcal, pneumococcal, COVID-19, and influenza	61	89.7	7	10.3
The meningococcal vaccine is mandatory for Hajj workers	25	36.8	43	63.2
The pneumococcal vaccine is mandatory for Hajj workers	22	32.4	46	67.6
The COVID-19 vaccine is mandatory for Hajj workers	33	48.5	35	51.5
The influenza vaccine is mandatory for Hajj workers	33	48.5	35	51.5
Respiratory pathogens can lead to serious complications	62	91.2	6	8.8
Crowded environments increase the risk of respiratory infection transmission	66	97.1	2	2.9

Regarding the overall knowledge level of vaccination and infectious disease risk among non-healthcare workers and volunteers during the Hajj, more than half of the participants (n = 39; 57.4%) had a good level of knowledge, while 29 individuals (42.6%) had a poor knowledge level. 

Reasons behind vaccinations

Among those who reported receiving vaccines, the primary motivation was disease prevention during Hajj (n = 10; 90.9%). Other significant reasons included following health authority recommendations (n = 6; 54.5%) and confidence in vaccine effectiveness (n = 6; 54.5%). Fewer participants mentioned avoiding sick leave (n = 2; 18.2%), while only one participant (9.1%) reported not receiving any vaccination during the season. Among those who did not receive vaccines, the most commonly cited reason was uncertainty about whether vaccination was a requirement for the Hajj work (n = 28; 49.1%). Other barriers included having already received the vaccine during seasonal work (n = 18; 31.6%), fear of side effects (n = 16; 28.1%), lack of knowledge about where to get vaccinated (n = 14; 24.6%), time constraints (n = 13; 22.8%), scheduling conflicts (n = 13; 22.8%), and limited access to vaccines (n = 10; 17.5%). A small portion (n = 5; 8.8%) reported other unspecified reasons.

Beliefs and opinions

Most respondents rejected common vaccine-related misconceptions. A large proportion (n = 49; 72.1%) rejected the belief that vaccines cause autism, with only 2 (2.9%) agreeing and 17 (25.0%) remaining neutral. Similarly, 46 participants (67.6%) disagreed with the statement that vaccines contain harmful toxins, while a minority agreed (n = 3; 4.4%) or were neutral (n = 19; 27.9%). When asked whether vaccines cause more harm than good, 47 (69.1%) disagreed, but a notable 10 participants (14.7%) agreed, and 11 (16.2%) remained neutral, suggesting some hesitancy in this area. Regarding conspiracy beliefs, 49 participants (72.1%) disagreed that vaccines are part of a conspiracy, while 6 (8.8%) agreed and 13 (19.1%) were neutral.

Willingness to take vaccines

Stated willingness to receive vaccines before working during the Hajj season was considerably higher than observed uptake. The flu vaccine had the highest level of readiness, with 64 participants (94.1%). Readiness for the COVID-19 vaccine was also relatively high (n = 41; 60.3%), followed closely by the meningococcal vaccine (n = 39; 57.4%) and the pneumococcal vaccine (n = 38; 55.9%). When asked if they would take the vaccines if provided free of charge by their employer, 42 participants (61.8%) responded "yes," while 22 (32.4%) were uncertain, and 4 (5.9%) said "no." Similarly, if the vaccines were made mandatory by government authorities, 51 (75.0%) indicated they would comply, though a smaller portion remained hesitant (n = 13; 19.1%), and 4 (5.9%) were opposed. Employer recommendations were also influential, with 41 participants (60.3%) stating they would take the vaccines if advised by their employer, 22 (32.4%) responding "maybe," and 5 (7.4%) refusing. Finally, 40 participants (58.8%) agreed that vaccination should be a requirement for working during the Hajj season, although 19 (27.9%) were unsure and 9 (13.2%) disagreed (Table 3).

**Table 3:** Associations Between Participant Characteristics and Self-Reported Vaccine Uptake Among Non-healthcare Workers and Volunteers During Hajj (N = 68) p-Values represent exact probabilities derived from Fisher's exact test (2×2 comparisons) and the Fisher-Freeman-Halton extension (r×c comparisons). Fisher's exact test does not produce a chi-square or t-statistic; the exact p-value constitutes the test statistic. *p < 0.05.

Factor	Meningococcal Vaccine			Influenza Vaccine			Pneumococcal Vaccine		
	Yes (n)	Yes (%)	p-Value	Yes (n)	Yes (%)	p-Value	Yes (n)	Yes (%)	p-Value
Age (years)									
18-39	8	15.7	0.646	22	43.1	0.049*	7	13.7	0.588
40-60	3	21.4		9	64.3		3	21.4	
>60	0	0.0		3	100.0		0	0.0	
Gender									
Male	10	17.2	0.566	27	46.6	0.171	9	15.5	0.649
Female	1	10.0		7	70.0		1	10.0	
Nationality									
Saudi	10	18.9	0.257	29	54.7	0.144	9	17.0	0.319
Non-Saudi	1	6.7		5	33.3		1	6.7	
Work experience									
<5 years	2	25.0	0.769	2	25.0	0.085	2	25.0	0.631
5-10 years	5	14.7		15	44.1		4	11.8	
>10 years	4	15.4		17	65.4		4	15.4	
Chronic disease									
Yes	1	7.7	0.356	8	61.5	0.355	1	7.7	0.427
No	10	18.2		26	47.3		9	16.4	
Knowledge level									
Poor	1	3.4	0.014*	10	34.5	0.027*	1	3.4	0.024*
Good	10	25.6		24	61.5		9	23.1	

Factors linked to vaccination

Participants with a good knowledge level were significantly more likely to have received the meningococcal vaccine (25.6%) compared to those with a poor knowledge level (only 3.4%). Conversely, 96.6% of those with poor knowledge had not received the vaccine (p = 0.014).

Vaccine uptake increased with age. All participants aged 60 and above (100%) had received the influenza vaccine. Among those aged 40-60 years, 64.3% were vaccinated, compared to only 43.1% in the 18-39 age group (p = 0.049). Also, participants with good knowledge were significantly more likely to have received the vaccine (61.5%) than those with poor knowledge (34.5%). Conversely, 65.5% of those with poor knowledge had not received the vaccine (p = 0.027).

Participants with good knowledge were significantly more likely to have received the pneumococcal vaccine (23.1%) compared to those with poor knowledge (only 3.4%). In contrast, 96.6% of individuals with poor knowledge had not received the vaccine (p = 0.024).

## Discussion

The key preliminary finding of the study was the poor take-up rate of required vaccines, such as meningococcal (16.2%) and pneumococcal (14.7%), despite high awareness of infectious disease risk. COVID-19 and flu vaccines were used more commonly (70.6% and 50%, respectively). This might be explained by the post-pandemic effect and the fact that the COVID-19 vaccine was mandatory for public outings compared to pneumococcal or meningococcal vaccines.

For example, one Malaysian study of the Hajj pilgrims noted 28.6% and 25.4% influenza and pneumococcal vaccine coverage, respectively, due in part to limited access and a lack of clear information on vaccine requirements [8]. In this study, confusion on whether vaccines were mandatory or not was also cited as a reason for not having been vaccinated by many of the respondents. Similarly, research among Saudi and international pilgrims has shown that overseas pilgrims usually have high meningococcal coverage [10].

In a large global survey of 19 countries, Lazarus et al. [11] found that about 71.5% of respondents reported that they would take a COVID-19 vaccine if it were safe and effective, but just 48.1% would take it if their employer suggested it. Similarly, in our preliminary data, people were more likely to get vaccinated if the vaccine was free or mandatorily required, compared to on the grounds of employer suggestion alone.

A relevant study among university students in Saudi Arabia [9] found that the flu vaccine had previously been taken by just 30.5% of the students before the winter season. The most common barrier was the lack of physician recommendation, followed by fear of side effects and lack of awareness barriers, which were also commonly reported in our preliminary study. What stands out in both studies is the important influence of healthcare professionals and trusted sources on vaccine decision-making.

Other evidence also suggests these findings. For instance, a Saudi study among healthcare workers during the Hajj reported very high meningococcal vaccine uptake (over 80%) but extremely low influenza coverage (less than 10%), showing that mandatory requirements strongly influence behaviour [12]. Likewise, studies of European pilgrims found large variation in vaccine coverage depending on the country of origin and access to clear pre-travel health information, further highlighting that adherence is context dependent [13]. Reviews of barriers to vaccination in the Hajj settings also emphasize recurring issues such as cost, logistics, misinformation, and cultural hesitancy, which are consistent with the barriers identified in our sample [6].

Our findings are also aligned with the current evidence showing a clear relationship between knowledge and vaccination. In our research, the odds of vaccination for meningococcal, influenza, and pneumococcal diseases were greater in participants who had good knowledge. This is comparable to what was seen in Mallhi et al.'s study [9], where students who had higher knowledge scores were more likely to accept the flu vaccine.

This study has several limitations that must be acknowledged. First, convenience sampling via online platforms, including WhatsApp and X, introduced selection bias, as participants who are more digitally connected or health-conscious may have been overrepresented. Second, the study relied entirely on self-reported vaccination status, which cannot be independently verified and is susceptible to recall and social desirability bias. Third, the final sample of 68 participants did not reach the intended target, substantially limiting statistical power and the generalizability of findings to the broader population of non-healthcare Hajj workers. Fourth, the retrospective nature of data collection, conducted after the 2024 season, increases the likelihood of recall bias. Additionally, the analysis was limited to bivariate testing using Fisher's exact test; multivariate analysis was not performed due to the small sample size and the exploratory nature of the study, which further constrains the strength of any inferences drawn. These limitations collectively constrain the extent to which conclusions can be generalized, and findings should be interpreted as preliminary and hypothesis-generating.

## Conclusions

These preliminary findings suggest that vaccination adherence among non-healthcare Hajj workers and volunteers may be suboptimal, particularly for meningococcal and pneumococcal vaccines. The observed association between knowledge level and vaccine uptake indicates that educational gaps may contribute to low adherence in this group. Strategies such as improving health communication, facilitating vaccine access, and clarifying official requirements may be worth exploring in future interventions; however, given the exploratory design and small sample size of this study, these observations should not be interpreted as definitive conclusions. Further research with larger, more representative samples is needed before any policy-level recommendations can be drawn.

## Appendices

The following instrument was used for data collection.

Section 1: Demographics

1. What is your age?

• 18-29

• 30-39

• 40-49

• 50-59

• 60+

2. What is your gender?

• Male

• Female

3. What was your occupation during the Hajj work?

• Do you work as a healthcare worker?

(Please specify): ____________________

4. Do you have any chronic medical conditions?

(Check all that apply):

• Diabetes

• Asthma

• Heart Disease

• Other (please specify): ____________________

• None

Section 2: Vaccination Status

5. Did you receive the meningococcal vaccine as part of your preparation for Hajj work?

• Yes

• No

• Unsure

6. Did you receive the influenza vaccine as part of your preparation for Hajj work?

• Yes

• No

• Unsure

7. Did you receive the pneumococcal vaccine as part of your preparation for Hajj work?

• Yes

• No

• Unsure

8. Did you receive the COVID-19 vaccine as part of your preparation for Hajj work?

• Yes

• No

• Unsure

Section 3: Motivations for Vaccination

9. If you received any vaccines, what were your main reasons?

(Check all that apply):

• Following the health authority’s advice

• Preventing illness during Hajj

• Confidence in vaccine effectiveness

• Avoiding sick leave during work

• Other (please specify): ____________________

Section 4: Reasons for Not Receiving Vaccines

10. If you did not receive a vaccine, what were your reasons?

(Check all that apply):

• Uncertainty about whether vaccination is required for Hajj work

• Unaware of clinics that provide vaccinations

• Limited access to vaccinations

• Living in an area where vaccines are not available

• Lack of time to get vaccinated

• Vaccine availability conflicts with personal schedule

• Misconceptions about vaccines (check all that apply):

• Vaccines cause autism

• Vaccines contain harmful toxins

• Vaccines do more harm than good

• Vaccines are part of a conspiracy

• Other (please specify): ____________________
